# Intimate Partner Violence and HIV Prevention Among Sexual Minority Men: Protocol for a Prospective Mixed Methods Cohort Study

**DOI:** 10.2196/41453

**Published:** 2022-11-15

**Authors:** Erik D Storholm, Dan E Siconolfi, Glenn J Wagner, Wenjing Huang, Carrie L Nacht, Greg Sallabank, Jennifer K Felner, Joshua Wolf, Sarita D Lee, Rob Stephenson

**Affiliations:** 1 School of Public Health San Diego State University San Diego, CA United States; 2 RAND Corporation Santa Monica, CA United States; 3 School of Nursing University or Michigan Ann Arbor, MI United States

**Keywords:** intimate partner violence, cohort study, sexual minority men, HIV, sexually transmitted infections, pre-exposure prophylaxis, PrEP

## Abstract

**Background:**

Sexual minority men experience intimate partner violence (IPV) at rates similar to those reported by heterosexual women in the United States. Previous studies linked both IPV victimization and perpetration to HIV risk and seroconversion; however, less is known about the impact of IPV on HIV testing, sexually transmitted infection (STI) testing, pre-exposure prophylaxis (PrEP) uptake, and the persistence of PrEP use among sexual minority men experiencing IPV. Although prior work suggests that IPV may influence HIV prevention behavior, experiences of IPV are so highly varied among sexual minority men (eg, forms, frequency, and severity; steady vs casual partnerships; perpetration vs receipt; and sexual vs physical vs psychological violence) that additional research is needed to better understand the impact that IPV has on HIV risk and protective behaviors to develop more effective interventions for sexual minority men.

**Objective:**

This study aims to contribute to our understanding of the antecedents of IPV and the direct and indirect pathways between perpetration and receipt of IPV and HIV or STI risk behavior, STIs, and use of PrEP among sexual minority men experiencing IPV.

**Methods:**

This mixed methods study has 2 phases: phase 1 involved formative qualitative interviews with 23 sexual minority men experiencing IPV and 10 key stakeholders or providers of services to sexual minority men experiencing IPV to inform the content of a subsequent web-based cohort study, and phase 2 involves the recruitment of a web-based cohort study of 500 currently partnered HIV-negative sexual minority men who reside in Centers for Disease Control and Prevention–identified Ending the HIV Epidemic priority jurisdictions across the United States. Participants will be followed for 24 months. They will be assessed through a full survey and asked to self-collect and return biospecimen kits assessing HIV, STIs, and PrEP use at 0, 6, 12, 18, and 24 months. They will also be asked to complete abbreviated surveys to assess for self-reported changes in key study variables at 3, 9, 15, and 21 months.

**Results:**

Phase 1 was launched in May 2021, and the phase 1 qualitative interviews began in December 2021 and were concluded in March 2022 after a diversity of experiences and perceptions were gathered and no new ideas emerged in the interviews. Rapid analysis of the qualitative interviews took place between March 2022 and June 2022. Phase 2 recruitment of the full cohort began in August 2022 and is planned to continue through February 2024.

**Conclusions:**

This mixed methods study will contribute valuable insights into the association that IPV has with HIV risk and protective behaviors among sexual minority men. The findings from this study will be used to inform the development or adaptation of HIV and IPV prevention interventions for sexual minority men experiencing IPV.

**International Registered Report Identifier (IRRID):**

DERR1-10.2196/41453

## Introduction

### Background

Sexual minority cisgender men experience alarming rates of intimate partner violence (IPV). Rates of IPV victimization or perpetration range from 1 in 4 to 1 in 2 sexual minority men [1-4]. In the 2011 National Intimate Partner Violence Survey, 25% of sexual minority men reported lifetime physical victimization and 60% reported lifetime psychological victimization from intimate partners [5]. These rates are similar to those in other large samples of sexual minority men [6-12]. Estimated lifetime prevalence for receipt of IPV among sexual minority men ranges from 12% [13] to 45% [14] for physical forms, 2% [15,16] to 33% [14] for sexual forms, 28% [17] to 64% [15,16] for psychological forms, and 32% [18] to 78% [19] for any form of IPV. Perpetration of IPV is less studied and ranges from 8% [1] to 35% [20]. Among sexual minority men, IPV has been associated with minority race or ethnicity [18,21-23], less education [10], positive HIV status [10, 23,24], and being aged 15 to 24 years [6,25-27].

The overarching goal of the proposed study is to examine how IPV influences HIV risk behavior and contributes to gaps in engagement in the HIV prevention continuum at 3 specific points: HIV testing (awareness), pre-exposure prophylaxis (PrEP) uptake (uptake), and PrEP persistence (adherence or retention [28]). A recent cross-sectional study found associations between the use of PrEP and specific dimensions of IPV victimization and speaks to the need for greater attention to the multidimensional aspects of IPV as they relate to HIV prevention continuum engagement [29]. Forced sex and emotional forms of IPV were negatively associated with PrEP use; however, controlling forms of IPV were positively associated with PrEP use. The authors asserted the need for longitudinal research to better assess causality and understand the fluctuations that are likely to occur in IPV, HIV risk, and PrEP use over time [29].

Research has linked both IPV victimization and perpetration to HIV risk and seroconversion. A large longitudinal study of Latinx sexual minority men in Los Angeles, California, United States, found that any lifetime experience of IPV (perpetration or victimization) doubled the odds of HIV seroconversion during the study period [30]. In a meta-analysis of 17 studies with a cumulative total of 13,797 sexual minority men, experience of IPV was associated with being HIV positive and doubled the odds of having condomless anal sex [31]. Studies have also found both victimization and perpetration of IPV to be associated with condomless anal sex [4] among both casual and main sex partners [32-34]. IPV perpetration has also been associated with doubling the odds of condomless anal sex among sexual minority men [11]. There is preliminary evidence to suggest that IPV is associated with substance use among sexual minority men and that substance use in combination with IPV increases HIV risk. A study of 7844 sexual minority men found that IPV increased with stimulant and popper use, and substance use strengthened the association between IPV and being HIV positive [35].

### Theoretical Framework

**Figure 1:**
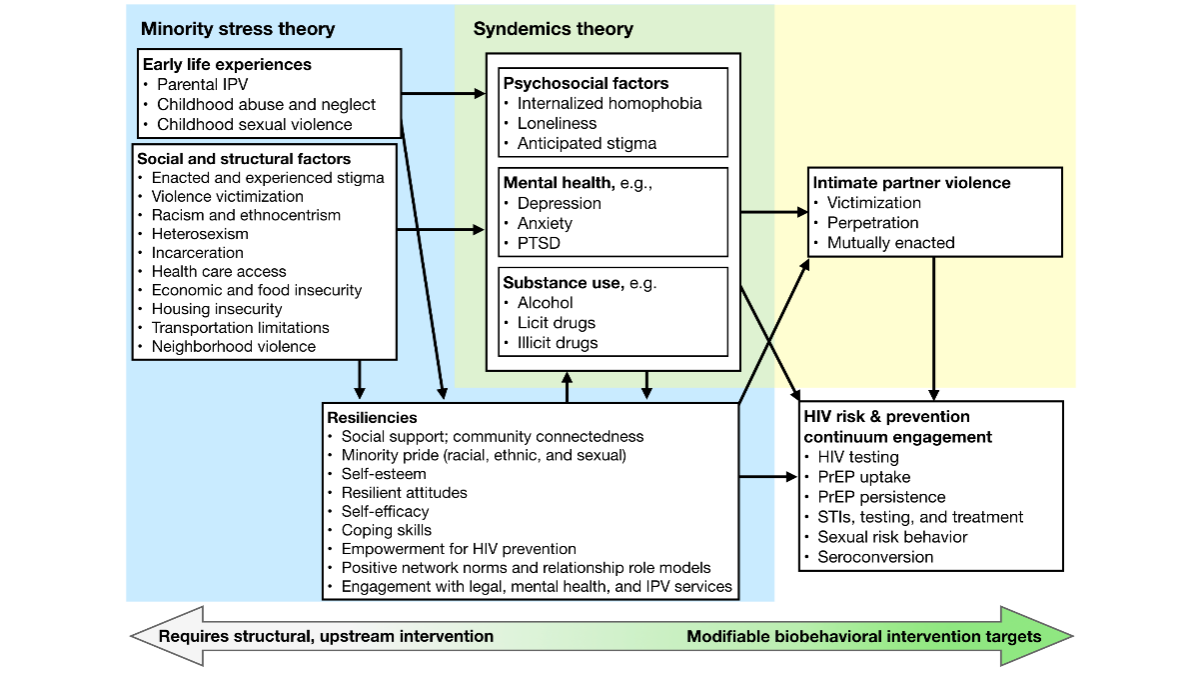
Theoretical framework. IPV: intimate partner violence; PrEP: pre-exposure prophylaxis; PTSD: posttraumatic stress disorder; STI: sexually transmitted infection.

Minority stress theory [36,37] and syndemics theory [38,39] provide lenses through which the cumulative effects of structural, social, and individual-level stressors on sexual minority men, including factors that increase the risk for IPV and HIV, can be examined. Both theories are widely used to understand antecedents of HIV disparities among sexual minority men and how health disparities overlap with potential synergistic effects [40-50]. A recent analysis of syndemics among Black sexual minority men showed an association between stress and depression symptoms, sexual compulsiveness, and experiencing IPV [51]. Several studies have identified correlations between internalized homophobia and perpetration of physical [15,16,51,52], sexual [52], and emotional or psychological IPV [52]. Homophobic discrimination and sexual orientation concealment are correlates of physical IPV perpetration [53]. Stephenson and Finneran [54] found that internalized homophobia, the experience of homophobia, and the experience of racism were positively correlated with the experience and perpetration of IPV among sexual minority men. Minority stress theory maintains that individuals who have marginalized identities, such as sexual minority men and racial or ethnic minorities, experience disproportionately burdensome degrees of stigma, discrimination, and victimization. These distal stressors occur alongside more proximal stressors such as anticipated rejection, shame, and negatively appraised experiences. As a result, sexual minority men are at risk for depression, substance use, and risk behaviors that may put them at risk for HIV [36,37,55]. Syndemics theory suggests that co-occurring psychosocial health conditions such as substance use, mental health problems, and violence are mutually reinforcing among marginalized minority populations [38,39,56]. Combined, the theories provide a framework (Figure 1) to understand IPV, HIV risk, and protective factors among sexual minority men, as well as potential intervention targets.

Although recent literature reveals IPV to be common and to have grave implications for sexual minority men’s health, there are critical methodological limitations and gaps in knowledge. Of the limited studies of IPV among sexual minority men, heterogeneous definitions and measures of IPV have led to unreliable and widely varied prevalence estimates [11,57]. Few studies have assessed psychological or emotional forms of abuse. Researchers have tended to use modified IPV measures developed for women without formal assessment of their appropriateness and psychometric characteristics among men or sexual minority men more specifically [11]. For bisexual sexual minority men, studies have poorly differentiated violence perpetrated by female partners versus male partners [58]. Recognizing that the IPV measure used in many studies, the Conflict Tactics Scale, was never validated for use with sexual minority men, Stephenson and Finneran [59] developed and validated a new scale, the IPV–Gay and Bisexual Men scale, to capture IPV, as experienced by men in same-sex relationships, in a large sexual minority men sample, which assesses IPV victimization and perpetration [60].

Very few studies have examined IPV perpetration among sexual minority men; most have focused on the receipt of IPV [11]. Little research has assessed mutually enacted or reciprocal IPV and associations with HIV risk and prevention outcomes among sexual minority men. Studies have neglected to differentiate IPV-like behaviors (eg, physical fights) that may in fact be self-defense. In addition, the preponderance of cross-sectional data (there are no published longitudinal studies of IPV among sexual minority men) and use of inconsistent recall periods (eg, 3 months to lifetime) greatly limit causal inferences about factors contributing to IPV and how IPV influences HIV risk behaviors.

These shortcomings have hampered the field’s basic understanding of the etiology, chronicity, severity, and escalation of IPV among sexual minority men, with consideration of the multiple forms of violence that exist (physical, sexual, and psychological). Together, this presents a knowledge gap regarding the 3 multidimensional aspects of IPV among sexual minority men (forms of violence, directionality of victimization, and frequency) and longitudinal trends among individuals or dyads. These 3 aspects are critical to intervention development.

Little is known about the mechanisms underlying the associations between IPV and the HIV prevention continuum, which undermines our ability to develop evidence-based interventions. Few studies have delved into the experiences and correlates of IPV for male-male couples. Previous, limited research suggests that stressors and triggers commonly acknowledged in the literature regarding heterosexual couples and IPV are also relevant to male-male couples (eg, substance use, jealousy, and financial stress) [58,61,62]. In addition to these, there are potentially distinct correlates among sexual minority men; for example, Finneran and Stephenson [58] found that these included discordance in HIV status, sexual disagreements (eg, positioning), a lack of sexual orientation outness, and competition to be the “alpha male” in a male-male relationship. Similarly, in their analysis of data from 403 sexual minority men in Atlanta, Georgia, United States, with main partners, Stephenson et al [63] found that IPV was less common among men with social networks that contained more gay-identified friends, highlighting the potential role of social support and peer modeling of healthy same-sex relationships. IPV in male dyads, while influenced by factors that are common to dyads of all genders and all types of couples, is shaped by factors specifically related to sexual orientation and the experience of being in a same-sex relationship.

### Objectives

The overall aim of the proposed study is to provide new knowledge of how perpetration and receipt of various forms of IPV (eg, physical, sexual, and psychological, as well as in the context of steady or casual intimate relationships) contribute to HIV risk, sexually transmitted infections (STIs), and HIV PrEP use among sexual minority men in the United States. Longitudinal research must unpack the global negative association of IPV with HIV risk, as well as how specific types of IPV may be associated with HIV risk and HIV prevention continuum engagement. This research is critical to the development of appropriate and effective HIV and IPV interventions for sexual minority men.

## Methods

### Study Design

This study represents a collaboration between researchers and staff at the San Diego State University, the University of Michigan, and the RAND Corporation. All survey assessments will be programmed and administered by the RAND Survey Research Group (SRG). Phase 1 research activities involved formative qualitative interviews with 23 sexual minority men who reported experienced or perpetrated IPV during the 12 months before the interviews and an additional 10 interviews with key stakeholders and providers of IPV-related services to sexual minority men to inform the subsequent cohort study to be completed in phase 2.

### Phase 1 Qualitative Data Collection

In phase 1, we conducted formative, in-depth interviews with 23 racially and ethnically diverse sexual minority men experiencing or perpetrating IPV in the past 12 months and 10 key stakeholders with experience providing services to sexual minority men experiencing or perpetrating IPV. The general purpose of these interviews was to inform the selection of measures and the development of additional questions for the phase 2 surveys and also inform methods and materials to be used for participant recruitment. To recruit a diverse sample of sexual minority men in terms of age and racial or ethnic identity, we used a combination of recruitment sources. We received direct referrals for participation from clinicians working directly with sexual minority men as part of an IPV prevention program for sexual and gender minorities. We also advertised the study through the social media page of a large urban lesbian, gay, bisexual, transgender, and queer service provider and by passing out flyers for the study in community spaces that sexual minority men were known to frequently visit (eg, gay bars, stores, and events). All phase 1 study activities took place in Los Angeles.

Interviews were conducted via video teleconference (through Zoom videoconferencing; Zoom Video Communications, Inc) with sexual minority men and focused on examining how participants’ lived experiences of IPV, both victimization and perpetration, may have directly or indirectly influenced their risk for HIV and STIs, their engagement in HIV testing, and their use of PrEP among other sexual health behaviors. For the stakeholder interviews, we purposely recruited participants from a wide range of professional roles interacting with and serving sexual minority men experiencing or perpetrating IPV, including counselors and program directors from an IPV-focused program, as well as additional mental and sexual health providers who work with sexual and gender minorities who have experienced IPV. Interviews with key stakeholders focused on the experiences of IPV victimization and perpetration that may be unique to sexual minority men, as well as any ways that they have noticed IPV affecting the sexual health of the sexual minority men they were working with. Interview probes specifically assessed for any relationships noticed between IPV and HIV and STI risks or engagement in prevention behaviors, including HIV and STI testing, PrEP uptake, and PrEP persistence, and elicited suggestions for what may be beneficial for future IPV and HIV prevention interventions focused on sexual minority men. Providers were also asked to provide feedback on the proposed recruitment methods and materials to be used in the recruitment of sexual minority men in the cohort study. All interviews were audio recorded and transcribed verbatim with identifying information removed to prepare for analysis. The phase 1 qualitative data analysis plan and how the data are being used to inform phase 2 study activities are described in the *Data Analysis Plan* section.

### Phase 2 Longitudinal Cohort Data Collection

**Figure 2:**
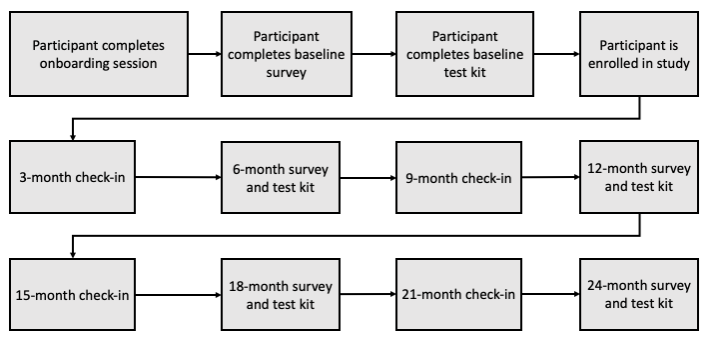
Phase 2 study flow diagram.

Each of the 500 participants who enroll in the prospective cohort study will be followed for 24 months. Each laboratory-confirmed HIV-negative cisgender sexual minority men participant will need to report being in a relationship with another cisgender sexual minority man for a minimum of 3 months at baseline and will also need to report residing in one of the Centers for Disease Control and Prevention (CDC)–identified Ending the HIV Epidemic (EHE) jurisdictions across the United States to be eligible for participation. Participants will be recruited through web-based advertising from across all the CDC-identified EHE jurisdictions. Figure 2 reflects the flow of each phase 2 study activity.

Participants will be followed for 24 months, with full study assessments at 0, 6, 12, 18, and 24 months and mini “check-in” assessments at 3, 9, 15, and 21 months to assess for changes in key study outcomes (IPV and engagement in HIV prevention). Participants will also submit self-collected dried blood spot (DBS) samples at 0, 6, 12, 18, and 24 months to test for the use of PrEP and for HIV seroconversion through mailed home test kits. Participants will also submit self-collected urine samples and self-collected rectal and pharyngeal swabs for the culture of gonorrhea and chlamydia at 0, 12, and 24 months through the same mailed home test kits. At each assessment, participants will receive up to 3 reminders based on their preference (either email or SMS text message, provided participants have consented to both) containing a link to the baseline survey at regular (eg, weekly) intervals during the survey window period. We currently plan to use a 28-day window but may expand or contract this window based on real-world completion rates.

This is a nonintervention cohort study. We will enroll 500 sexual minority men, allowing for 20% attrition, for a final sample of 400 sexual minority men at month 24. The eligibility criteria are listed in Textbox 1.

**Cohort study eligibility criteria.**

**Inclusion criteria**

- Assigned male sex at birth and currently identifying as man (cisgender male)
- Aged 18 to 45 years
- Reporting currently being in a relationship with a cisgender man lasting at least 3 months; a relationship is defined as “Do you have a primary male partner, that is, someone you feel emotionally, romantically committed to above others?”
- Residing in one of the Centers for Disease Control and Prevention Ending the Epidemic priority jurisdictions
- Able to provide at least two means of contact for follow-up
- Not currently enrolled in an HIV prevention study
- Having self-reported HIV-negative serostatus at baseline (status confirmed via home test kit mailed to laboratory)
- We may stratify eligibility as needed to ensure that at least 60% of participants report past-year intimate partner violence at baseline
- We will stratify as needed to ensure at least 35% of the sample identifies as Black or African American and at least 35% identifies as Hispanic or Latinx

**Exclusion criteria**

- Not assigned male sex at birth
- Aged <18 years or >45 years at enrollment
- Partnered <3 months or currently unpartnered
- Living outside of the Centers for Disease Control and Prevention Ending the Epidemic priority jurisdictions
- Self-reporting HIV-positive status or is laboratory determined to be HIV positive at baseline
- Expressing unwillingness to complete regular surveys during informed consent
- Expressing unwillingness to provide biospecimens with home testing kits during informed consent
- Expressing unwillingness to provide partner contact information (to allow us to screen for dyads)
- Individual’s romantic partner is already enrolled in the study (we will not enroll dyads)

### Recruitment

Investigators and research staff from San Diego State University and the University of Michigan will be responsible for all recruitment activities. The study will enroll participants who reside in one of the CDC-defined EHE priority jurisdictions. The “Ending the HIV Epidemic: A Plan for America” was launched by the US Department of Health and Human Services to reduce new HIV infections by 90% by 2030 by leveraging critical scientific prevention strategies in specified high-incident geographic locations [64]. The proposed activities will recruit participants from the counties, territories, and states included in the EHE plan. To recruit a sample from the CDC-identified EHE jurisdictions, we will use paid advertising on social media and dating or hook-up apps targeted to end users in the specified EHE locations (Textbox 2).

**Figure 3:**
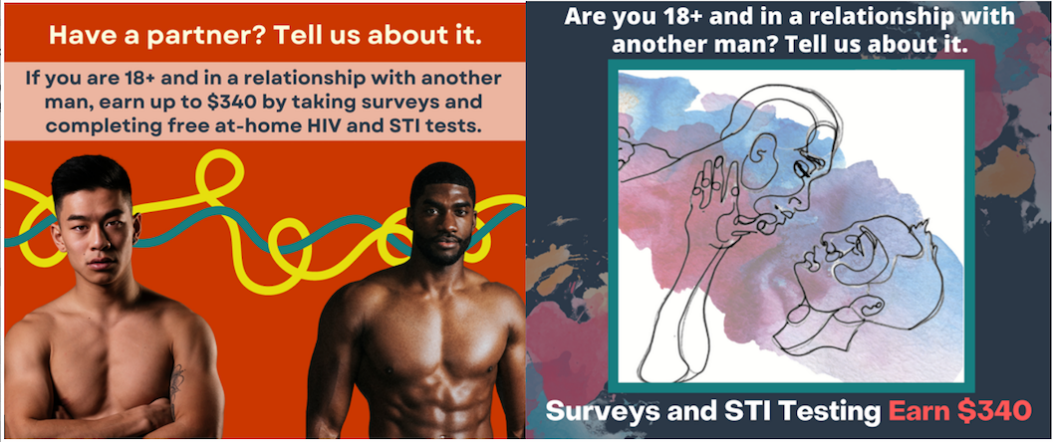
Sample cohort study advertisements. STI: sexually transmitted infection.

Figure 3 illustrates 2 sample advertisements for the study. We will use a combination of advertisements on the social networking websites Facebook and Instagram, as well as on mobile gay dating or hook-up apps (eg, Scruff, BarebackRT, Jack’d, and Grindr) to promote the study and recruit study participants. Web-based advertisements will show a variety of institutional review board–approved visual representations of sexual minority men across a range of races or ethnicities or will just feature institutional review board–approved call-to-action text and the study logo. Recruitment materials will highlight the eligibility criteria for currently being in a relationship and will state that this is a study focused on men’s health and relationships. Importantly, recruitment materials will not mention IPV for participant confidentiality and safety. The websites or apps we recruit from will not have access to the screener data.

Advertisements will be targeted at adult (aged 18-45 years) sexual minority men in the EHE jurisdictions. We will be using the social media platforms Facebook and Instagram, as well as the sexual networking apps mentioned above. Men with partners may have relationship agreements that allow for outside sex partners, hence, the decision to enroll through sex-seeking apps.

Men who click on the web-based or app-based advertisements will be shown a brief introduction script that describes the study. They will then be directed to a consent form (to be screened for eligibility) and complete a short demographic and behavioral eligibility survey form. Screening data will be accessible to study staff only.

Participants who are eligible based on the screener survey will be contacted by a study research coordinator to schedule their 30-minute virtual onboarding orientation session, during which the research coordinator will provide a thorough overview of all research activities and timelines, go over the biological specimen collection instructions, review an informed consent form, and collect the participant’s consent (through electronic signature) to participate in the cohort study.

**Centers for Disease Control and Prevention–identified Ending the HIV Epidemic states, counties, and territories.**

**States**

- Alabama, Arkansas, Kentucky, Mississippi, Missouri, Oklahoma, and South Carolina

**Counties**

- [Arizona] Maricopa; [California] Alameda, Los Angeles, Orange, Riverside, Sacramento, San Bernardino, San Diego, and San Francisco; [Florida] Broward, Duval, Hillsborough, Miami-Dade, Orange, Palm Beach, and Pinellas; [Georgia] Cobb, DeKalb, Fulton, and Gwinnett; [Illinois] Cook; [Indiana] Marion; [Louisiana] East Baton Rouge Parish and Orleans Parish; [Maryland] Baltimore City, Montgomery, and Prince George’s; [Massachusetts] Suffolk; [Michigan] Wayne; [Nevada] Clark; [New Jersey] Essex and Hudson; [New York] the Bronx, Kings, New York, and Queens; [North Carolina] Mecklenburg; [Ohio] Cuyahoga, Franklin, and Hamilton; [Pennsylvania] Philadelphia; [Tennessee] Shelby; [Texas] Bexar, Dallas, Harris, Tarrant, and Travis; and [Washington] King

**Territories**

- Puerto Rico’s San Juan Municipio and Washington DC

### Baseline Survey

Once a participant has completed the onboarding interview and consented to participate in the cohort study, the research coordinator will complete a study enrollment form to import the participant’s contact information and key data elements into the RAND SRG–developed Record Management System, which will then trigger a unique link to the baseline survey (programmed in Forsta) to be sent to the participant’s email address. The baseline survey has been informed by the phase 1 qualitative interview data and will include questions from the following domains: demographic characteristics; IPV, including *physical, sexual, emotional, financial, monitoring, stalking,* and *HIV prevention–specific* forms of IPV; mental health; relationship and partner; sexual behavior and agreements; experiences of stigma and discrimination; structural vulnerabilities; social support; spirituality and religiosity; HIV testing; PrEP uptake; PrEP persistence; STIs; and sexual risk behavior. Baseline measures are presented in Textbox 3.

Participants who do not complete the baseline survey during the survey open window will be removed from the study unless they contact the study team to request more time.

**Baseline survey measures.**

**Intimate partner violence (IPV)**

- Modified IPV–Gay and Bisexual Men scale (62 items assessing IPV among sexual minority men, including physical, sexual, psychological, and monitoring or stalking IPV) [59] and adapted financial control items [65]
- Disclosure of IPV experiences to or by others [66-68] (some questions adapted from the original version)
- Help-seeking behaviors and receipt of IPV services (some questions adapted from the original version)
- IPV victimization stigma and shame [69]
- IPV perpetration stigma and shame [70]
- Perceived prevalence of IPV [66-68] (some questions adapted from the original version)

**HIV prevention engagement**

- HIV testing [71] (self-report and medical record confirmation)
- Sexually transmitted infections testing and diagnosis [71] (self-report and biomarker)
- Sexual behavior and condomless sex (self-report)
- HIV status (self-report, medical record confirmation, and biomarker)
- Pre-exposure prophylaxis (PrEP) uptake and PrEP persistence (self-report and biomarker)
- Perceived PrEP adherence (self-report)
- Reasons for not using PrEP or stopping PrEP [66-68] (self-report)
- PrEP modality acceptability [72] (self-report)
- Long-acting injectable PrEP acceptability
- PrEP stigma [73] (self-report)
- PrEP use [71]

**Demographics**

- Age
- Race
- Ethnicity
- Nativity (United States–born)
- Employment status [74]
- Employment precarity [75]
- Financial well-being [76]
- Educational attainment [74]
- Food insecurity [77]
- Sexual orientation [74]
- Housing status and housing instability [78]
- Recent homelessness [79]
- Housing precarity [74]
- Gender identity [75] (planned for 6-month follow-up assessment)

**Health status and health care**

- Self-rated health [80]
- Insurance coverage [81]
- Physical health care use [82]
- Behavioral health care use and perceived unmet need [83]
- Current health conditions (planned for 6-month follow-up assessment)

**Partner and relationship characteristics (reported by index participant)**

- Relationship status
- Relationship characteristics (type; duration and history of separations)
- Marital status
- Cohabitation
- Partner demographics (race, ethnicity, gender, sexual orientation, educational attainment, age, and HIV status)
- Financial reliance
- Partner PrEP use or HIV treatment status or viral suppression
- PrEP conversations [66-68] (some questions adapted from the original version)
- Partner support for taking PrEP [66-68] (some questions adapted from the original version)
- Inclusion of other in self [84]
- Sexual agreements (type and adherence)
- Relationship power balance and decision-making [85]
- Communication patterns [86]
- Social support from partner [87]
- Relationship role models (regardless of relationship status)

**Early-life and childhood experiences**

- Childhood violence and abuse and mistreatment by adults [88] (planned for 6-month follow-up assessment)
- Victimization and bullying [89] (planned for 6-month follow-up assessment)

**Social and structural factors**

- Incarceration (lifetime and recent) [71]
- Experienced discrimination (racial or ethnic, sexual orientation, gender expression, and other characteristics) [90]
- Perceived neighborhood safety [91]
- Recent exchange or transactional sex [71]

**Mental health**

- Depressive symptoms [92]
- Loneliness [93] (planned for 6-month follow-up assessment)
- Posttraumatic stress disorder [94]
- Nonsuicidal self-injury [95] (planned for 6-month follow-up assessment)

**Substance use and abuse**

- Alcohol use [96]
- Illicit and licit substance use [97]

**Psychosocial factors**

- Internalized homophobia [98,99]
- Gay-related stigma [100]
- Racial and ethnic identity devaluation [101]
- Masculinity ideals and attainment
- Conformity to hegemonic male norms [102]
- Anticipated stigma (global demographics) [103] (some questions adapted from the original version)

**Resiliency factors**

- Lesbian, gay, bisexual, transgender, and other community affiliation [104]
- Global resiliency traits [105]
- Perceived social support (eg, emotional and instrumental) [87]
- Coping self-efficacy [106]
- Global self-esteem [107] (planned for 6-month follow-up assessment)

**Miscellaneous**

- Willingness to be contacted for future studies
- Survey satisfaction [108]
- Enrollment in other sexual minority men’s health studies

### Baseline Biospecimen Sample Collection

After the baseline survey has been completed, the participant’s provided mailing information is transferred to Molecular Testing Labs (MTL) through MTL’s application programming interface. The RAND SRG will assign a unique order ID to each specimen collection kit to inform MTL to mail the participant the specimen collection kit through US Postal Service Priority Mail. The collection kit will contain instructions and materials for collecting and returning the biospecimen samples. Packaging will be plain and discreet. After the participant has received their kit and collected their sample, they will return the samples using a prepaid mailer. Participants will be given 2 weeks from the date of kit delivery to collect their samples and return them in the mail for processing. They will receive up to 6 reminders through SMS text messages or email. After 4 weeks, participants who have not returned their baseline samples will be designated as having decided to not collect their samples and will be stopped from further study participation. In addition, those who screen HIV positive (at baseline) will not be enrolled in the full cohort study.

### Biospecimen Sample Collection Procedures

DBS specimens will be collected to detect the presence of HIV at baseline and 12- and 24-month assessments. DBS specimens will also be used to confirm reported use of PrEP at 0-, 6-, 12-, 18-, and 24-month assessment points when participants self-report taking PrEP (survey). The DBS procedure involves using a lancet to prick one’s fingertip, and 3 to 6 drops of blood are applied to each of the 5 circles on a DBS collection paper card. We will also collect self-administered urethral gonorrhea and chlamydia testing at baseline and 12- and 24-month assessment visits through the nucleic acid probe of urine specimens. Participants will provide a urine sample of 30 mL to 50 mL from the initial urine stream in a collection cup.

To complement the urine-based urethral samples, we will also collect self-administered rectal and pharyngeal swabs for gonorrhea and chlamydia testing at baseline and 12- and 24-month assessment visits. Briefly, participants will swab each site using provided collection swabs and place the swabs into transport tubes for processing. All survey and biospecimen procedures will take place according to the schedule presented in Table 1.

**Table 1:** Schedule of survey and biospecimen collection by time point^a^.

Outcome		Baseline- assessment	6-month assessment	12-month assessment	18-month assessment	24-month assessment
**Primary**						
	HIV testing behavior	Survey	Survey	Survey	Survey	Survey
	PrEP^b^ uptake	Survey+DBS^c^	Survey+DBS	Survey+DBS	Survey+DBS	Survey+DBS
	PrEP persistence	Survey+DBS	Survey+DBS	Survey+DBS	Survey+DBS	Survey+DBS
	STIs^d^ (CT^e^ and GC^f^)	Survey+culture	Survey	Survey+culture	Survey	Survey+culture
**Secondary**						
	Sexual risk behavior	Survey	Survey	Survey	Survey	Survey
	HIV seroconversion	Survey+DBS	Survey	Survey+DBS	Survey	Survey+DBS

^a^Brief assessments of relationship changes, intimate partner violence exposure, and self-reported HIV prevention continuum and STI outcomes at 3, 9, 15, and 21 months (not shown).

^b^PrEP: pre-exposure prophylaxis.

^c^DBS: dried blood spot.

^d^STIs: sexually transmitted infections.

^e^CT: chlamydia.

^f^GC: gonorrhea.

### Laboratory Testing and Follow-up

Samples will be tested by MTL for HIV, PrEP, and STIs (gonorrhea and chlamydia). In the event that MTL finds the quality of the sample to be insufficient, participants will be contacted by their preferred method of communication (either email or SMS text message) by the study team to request a second sample collection. MTL will mail a second kit, and the participant will re-collect the sample and mail it back to MTL.

Participants who have a reactive HIV or STI test will be contacted by study staff to let them know that they have received a preliminary positive test result and will be told that they need to schedule a visit with a local provider to confirm the result and be linked to care for treatment. Participants will be instructed that the laboratory test is for research purposes and that they should seek confirmation from their physician. Study staff will offer to help participants locate a local provider or clinic should they request assistance.

### Study Task Reminders and Participant Retention

The study will use multiple platforms to maintain the participant database, program the web surveys, and circulate email and SMS text message communications. Participant information, survey response data, test results, and other administrative data are maintained in a secure, encrypted database. Most email-based and SMS text message–based study communications (eg, survey notifications, reminders to complete a survey, and reminders to return a biospecimen kit) are automated, using scheduling criteria and prewritten templates that use a conversational tone and accessible reading level. For participants in danger of missing a study task (ie, within 7 days of a task deadline, such as a survey window closing), study staff will contact participants individually by telephone, email, or SMS text message.

### Compensation

Participants will have the potential to earn up to US $340 as remuneration (in the form of e-gift certificates) for participation in this study. Each survey time point includes US $25 as remuneration, each returned biospecimen kit includes US $25 as remuneration, and check-in surveys include US $10 as remuneration. Participants who complete all primary study tasks (full survey time points and biospecimen kits) will also receive a US $50 bonus at the conclusion of their follow-up.

### Data Analysis Plan

The analyses described in the following sections describe the analyses for phase 1 and our preliminary analysis plan for phase 2, specified in advance. Although the focal outcomes will remain the same, the specific analytic methods may change based on data (eg, feasibility of a proposed model, given the data distributions), evolution of the research questions (eg, based on advances in the field during the 2-3 years of data collection), and statistician input. We may also pursue additional analyses on secondary outcomes of interest.

### Phase 1 Qualitative Data Analysis

To inform the selection of measures, development of any additional items, and inform the development of recruitment materials and methods for phase 2 of the study, members of the study team reviewed and discussed analytic memos written by interviewers after each interview and memos written by coders upon reading the verbatim transcripts [109,110]. This resulted in the identification of preliminary thematic patterns that informed the baseline survey so that the phase 2 cohort study was able to launch in August 2022.

In June 2022, we began the process of rigorously confirming the preliminary findings that informed the baseline survey using an applied thematic analysis approach to identify all key patterns across our 2 sources of qualitative data [111]. This process will lead to the publication of our qualitative findings and will further inform future survey assessments (eg, 6-month assessments and 12-month assessments). Data (transcripts) are being organized and coded in NVivo qualitative data analysis software (QSR International) [112] using a codebook of deductive (ie, a priori, codes), as well as inductive codes grounded in the data. Coders tested iterations of the codebook on a series of transcripts over time to identify needed updates (ie, adding new codes, deleting redundant codes, and refining code definitions). To formally assess intercoder agreement, the coders double-coded >10% of the transcripts, discussing coding discrepancies and updating the codebook in real time. After multiple rounds of intercoder agreement assessment (final Cohen κ>0.80, suggesting sufficient reliability in coding), the codebook was finalized, and the coders began independently coding the transcripts [113].

Once all qualitative data have been coded, we will develop code summaries (high-level summaries of excerpts for individual codes), which will be reviewed and discussed by members of the research team with attention to repetition, frequency, salience of ideas, and meaning within context across interviews. Multiple rounds of group-level discussion, rereading of code summaries and specific coded excerpts, visually mapping relationships between key themes and subthemes, and triangulation across the sexual minority men and key stakeholder interviews [114] will inform final theme development [111,115] (Felner, JK, unpublished data, March 2022) and, in turn, the remaining phase 2 study activities.

### Phase 2 Quantitative Data Analyses

Phase 2 analyses will examine the robustness of our measures and our sample. First, we will assess the psychometric properties of all measures. Second, Wilcoxon rank sum and chi-square tests will be performed on baseline and follow-up variables to test for differences between dropouts and completers. Statistical adjustments will be used to correct for effects of attrition [116]. Standard multiple imputation techniques [117] could be used to account for missing data, such as imputing covariates using sequential Bayesian additive regression trees (using the R package *sbart*), which is nonparametric and avoids assuming that covariates are related [118]. Before building more elaborate latent curve models (LCMs), we will first explore relationships among our measures using correlations, regressions, and simple structural equation models (SEMs).

In phase 2, we will model multiple developmental trajectories of 5 waves of data using LCMs. We will examine three developmental trajectories: (1) the trajectory of the predictor, IPV; (2) the trajectory of the primary outcomes, HIV prevention continuum engagement and STI diagnoses; and (3) the trajectory of the secondary outcomes, risky sexual behavior (condomless anal sex) and seroconversion. The predictor trajectory is defined by repeated measures of IPV. As LCM allows for the estimation of multiple trajectories at the same time, we are also modeling a parallel process of HIV prevention continuum engagement. These primary outcomes are based on repeated measures of 3 binary indicators: HIV testing, PrEP uptake, and PrEP persistence. Although engagement in these HIV prevention continuum indicators has been described as discrete steps [28], the approach we propose is innovative as we will build measurement models based on these 3 indicators. From a factor analytic perspective, these are dichotomous indicators used to define a single latent HIV prevention continuum factor (*f*). With 5 waves of data, the repeated measures of the same latent variable are represented by *f1* to *f5* in the LCM. The developmental trajectory of HIV prevention continuum engagement will then be based on the latent variables *f1* to *f5*. In a similar fashion, we can model the relationship between the predictor trajectory and the trajectory for STI diagnoses (primary outcome) and condomless anal sex and seroconversion (secondary outcomes).

We will explore trajectory patterns to assess whether growth is linear or nonlinear. If it is nonlinear, we will explore quadratic effects or piecewise developmental patterns. To our knowledge, modeling the developmental trajectory of a latent HIV prevention continuum factor using multiple indicators is novel. Furthermore, we have multiple parallel developmental processes (IPV, prevention continuum, STIs, condomless sex, and seroconversion) in the same growth model.

We hypothesize that resilience factors (eg, coping skills, greater social support, and positive role models) will mediate (or moderate) the relationship between IPV and HIV risk and strengthen the association between IPV and HIV prevention continuum outcomes. We also hypothesize mediating effects of potential risk factors (eg, substance use, poorer mental health, and incarceration, as well as partner- and relationship-level factors). Such potential mediating effects can easily be incorporated into SEMs. Given the complicated LCM setup with multiple developmental trajectories, we anticipate challenges in adding moderators directly into the growth model.

A way of overcoming these challenges is to use multiple group analysis. By comparing relationships between the predictor trajectory and the outcomes trajectories across different groups, multiple group analysis allows us to (1) test different assumptions about group equality [119] and (2) build appropriate models for different, heterogeneous subpopulations. Given the diversity of life experiences in our sample, the relationships between IPV and HIV prevention continuum engagement may vary based on these diverse behaviors and psychosocial states. Moreover, our inclusion of resilience lends itself to the development of strength-based interventions where the buffering effect of resilience is evidenced in sexual minority men, according to Storholm et al [120]. Various time-varying and invariant covariates can also be added to the LCM to explore the effects of the characteristics of sexual minority men, their intimate partners, and their relationship dynamics. We will use Mplus (Muthén and Muthén) for these analyses [121]. We will test different moderating and mediating effects, focusing on the different risk and protective factors, while controlling for demographic and socioeconomic characteristics. Alternative models will be compared using a set of model fit indices, including root mean square error of approximation, Tucker-Lewis index, and various fit statistics, as described by Jöreskog and Sörbom [122].

### Ethics Approval

All study protocols and procedures have been approved by the San Diego State University Institutional Review Board (phase 1 protocol number HS-2021-0054 and phase 2 protocol number HS-2022-0094). All procedures are in accordance with the ethical standards of the institutional and national research committees and with the 1964 Helsinki declaration and its later amendments or comparable ethical standards. Informed consent was or will be obtained from all participants included in the study.

### Data Sharing Plan

This project does not exceed the US $500,000 cap set by the National Institutes of Health in any project year. However, based on the importance of the data, we encourage collaborations with interested investigators. Data will be made publicly available through publication in peer-reviewed journals, public seminars, and invited lectures and conference presentations. After 2 years of publication of the main findings of the study, we will consult with the San Diego State University’s Human Research Protection Program about how to securely make data available in the form of an electronic database for researchers who successfully complete a registration process. Any shared data will be deidentified and will not contain any direct or indirect identifiers. As part of the registration process, users must complete a data sharing agreement that outlines the conditions of use governing access to the public release data, including restrictions against attempting to identify study participants, destruction of the data after analyses are completed, reporting responsibilities, restrictions on redistribution of the data to third parties, and proper acknowledgment of the data resource. The data sharing agreement will include a commitment to using the data only for research purposes, a commitment to securing the data using appropriate computer technology, and a commitment to destroying or returning the data after analyses are completed. Users must submit brief proposals regarding the intended use of the data; the study team will determine the scientific soundness of the proposal as part of the decision to allow the researchers to access the public use data set. Users must be monitored by an approved human subjects board.

## Results

This study was funded in March 2021 by the National Institute of Mental Health (R01MH126762). The study was launched in May 2021, and phase 1 interviews began in December 2021 and concluded in March 2022. Rapid analysis of the qualitative interviews took place between March 2022 and June 2022. Phase 2 recruitment of the full cohort began in August 2022 and is planned to continue through February 2024.

## Discussion

### Principal Findings

This study seeks to conduct a multidimensional, longitudinal assessment of IPV and HIV prevention continuum outcomes among sexual minority men. Unlike most studies that have only focused on physical contact forms of abuse, we will also assess forms of IPV that involve coercive control and psychological abuse. We hypothesize that IPV will have a deleterious overall impact on HIV prevention continuum engagement and that the pathways between IPV and HIV prevention continuum engagement will be mediated by individual (eg, internalized homophobia, mental health, and substance use), interpersonal (eg, social support, relationship characteristics, and gender norms), and structural-level (eg, poverty, incarceration, health care access, and neighborhood violence) factors. Our measurement of IPV will include measurement of both the receipt and perpetration of IPV among sexual minority men, unlike prior research that has largely neglected IPV perpetration. Our assessment of the chronicity of IPV is also novel, as is the type of relationships in which it occurs. Most of the research has assessed either lifetime or recent (eg, past-year) experiences of IPV, which precludes analysis of chronicity, spacing of episodes, or escalation or waning of IPV.

We will use a longitudinal design for temporality to better understand the potential mechanisms between IPV and HIV risk and protective factors. We will use SEM to assess the intersectionality of multiple syndemic factors made possible with methods that we have refined over several previous studies of sexual minority men. This will allow us to explore the unique and common effects of different kinds of stigmas and supportive factors on HIV risk and HIV prevention outcomes. A longitudinal approach allows us to assess temporality with regard to associations between IPV and HIV prevention continuum engagement and heterogeneous phenotypes therein; for example, we will be able to differentiate sexual minority men who experience simultaneous IPV and poor HIV prevention continuum engagement from men whose IPV experiences precede worsening HIV prevention continuum engagement or HIV risk behaviors. We will also be able to assess whether HIV prevention continuum engagement is associated with waning IPV over time, perhaps as sexual minority men avail themselves of wraparound services linked to HIV prevention continuum services. Longitudinal assessment also allows us to assess how mediators and moderators such as mental health problems, psychosocial factors, and resiliency factors change over time in this context. Although a longitudinal design is critical, it is also necessary to have a large enough sample (for statistical power) and a long enough follow-up period to adequately examine the complexity of the dynamics in play with IPV and HIV risk among sexual minority men in multiple types of relationships, all of which necessitates and is accomplished by the proposed research.

In addition, we will examine resiliencies and risk factors to better understand underlying mechanisms and identify modifiable intervention targets. Most studies have focused on risk factors. Much less is known regarding the vitality of protective factors in the health of sexual minority men. To design IPV and HIV prevention interventions for sexual minority men, it is vital that we also examine protective factors. Our study may be the first to elucidate the potential effects of resiliencies such as coping skills, social support, and sexual minority pride in the constellation of risk factors between IPV and poor HIV prevention continuum engagement. Understanding the important role of these protective factors is vital to the development of innovative strengths-based interventions.

### Limitations and Strengths

A handful of limitations could affect this study and are important to acknowledge. First, documentation of IPV, both in terms of victimization and perpetration, will rely on self-report. As such, there may be underreporting of perpetration because of fear of possible legal consequences. There may also be underreporting of victimization because of potentially socially desirable responding and social norms that shame male survivors of IPV. To minimize these concerns, in-depth interviews will provide insights into how best to assess both victimization and perpetration in the context of same-sex male relationships. In addition, by conducting a prospective investigation, we will be uniquely positioned to examine how current experiences of IPV influence gaps in HIV prevention continuum outcomes prospectively. Second, the measurement of perceived social support, a potential buffer between IPV and IPV-associated HIV prevention continuum gaps, is based on the egocentric assessment of the degree to which individuals perceive receiving social support from a range of social network members (eg, peers, family members, and coworkers). However, obtaining egocentric-level data on multiple forms of perceived social support using a validated measure is a common and widely accepted approach.

The proposed study will be designed and implemented with a high degree of scientific rigor and has the potential for significant public health impact. First, we have a core team with expertise in the design and conduct of prospective cohort studies in IPV, HIV risk, and HIV prevention continuum outcomes among sexual minority men, as well as in advanced statistical analyses. Second, we will measure exposures, moderators, and outcomes at multiple time points in this prospective cohort study. Third, we will be conducting this study in the EHE-identified HIV high-incidence jurisdictions.

### Dissemination Plan

The research team will collaborate with key community stakeholders to review and validate the data and findings and make recommendations for intervention development or adaptation. We will also review interventions from the CDC compendium of evidence-based interventions to examine where content that addresses IPV may be integrated. The aim is not necessarily to develop an intervention in the time frame of this study but rather to use the data to provide strong evidence for the forms, types, and content of new interventions or adaptations to existing interventions. It is likely that this planning phase will lead to the development of an intervention that we will test in the next phase of our ongoing research program. Dissemination of findings will take place primarily through the publication of scientific manuscripts and stakeholder-oriented research briefs; professional conference presentations; and community-focused meetings and presentations with stakeholder groups at local, regional, state, and national levels. We will maximize the impact of our dissemination efforts through best-practice approaches such as framing the presentation of results in ways that highlight the relevance to various stakeholder audiences and by working with trusted intermediary organizations.

### Conclusions

This longitudinal cohort study will provide the data needed to better understand the direct and indirect ways in which IPV affects HIV risk and prevention behaviors. With the goal of providing concrete recommendations for intervention development, we believe that this is the first study to provide the needed science to develop and promote interventions to reduce the harms associated with both IPV and HIV risk or HIV prevention continuum outcomes. These findings will fill a critical gap in our efforts to reduce HIV-related disparities as part of the National Institutes of Health Strategic Plan for HIV and HIV-Related Research.

## Abbreviations

CDC: Centers for Disease Control and Prevention
DBS: dried blood spot
EHE: Ending the HIV Epidemic
IPV: intimate partner violence
LCM: latent curve model
MTL: Molecular Testing Labs
PrEP: pre-exposure prophylaxis
SEM: structural equation model
SRG: Survey Research Group
STI: sexually transmitted infection
